# Development of a standardized ovine aortic stenosis model: a pathomimetic platform for TAVI evaluation and durability testing

**DOI:** 10.3389/fcvm.2025.1663130

**Published:** 2025-11-25

**Authors:** Yan Zhu, Nana Qin, Jie Hou, Baoyin Li, Fangxu Liu, Xin Jiang, Zhuo Chen, Xiongtao Lin, Xiaohang Tong, Xufeng Wei, Dejian Li, Mengsi Hu

**Affiliations:** 1 Department of Cardiovascular Surgery, General Hospital of Northern Theater Command, Shenyang, China; 2 PerMedos Medical Technology Co., Ltd., Shanghai, China; 3 Postdoctoral Programme of Taizhou Pharmceutical Innovation Service Center, Nanjing Medical University, Taizhou, China; 4 Department of Cardiology, Wuxi Mingci Cardiovascular Hospital, Wuxi, China; 5 Department of Nephrology, Shandong Provincial Hospital Affiliated to Shandong First Medical University, Jinan, China; 6 Department of Nephrology, Shandong Provincial Hospital, Cheeloo College of Medicine, Shandong University, Jinan, China; 7 Hisense Postdoctoral Research Station, Qingdao, China

**Keywords:** aortic stenosis, TAVI, sheep model, hemodynamics, calcification

## Abstract

**Background:**

Transcatheter aortic valve implantation (TAVI) has gained widespread clinical acceptance owing to its minimally invasive approach and enhanced postoperative recovery. This study developed a standardized ovine aortic stenosis (AS) model through surgical implantation of a circular bioengineered annular stent in the aortic root, creating a reproducible pathomimetic platform for TAVI evaluation. Following hemodynamic stabilization, TAVI procedures were performed to systematically assess mid-to-long-term valve functionality and calcification progression.

**Methods:**

AS model was established in 11 sheep using extracorporeal circulation assistance technology. Following hemodynamic stabilization (2–4 weeks), TAVI was performed. The modeled sheep were divided into two groups to monitor valve conditions at 3 months (*n* = 6) and 6 months (*n* = 5), respectively. Additionally, a non-modeled control group was established, with valve conditions assessed at 3 months (*n* = 2) and 6 months (*n* = 1). Preoperative ultrasound data, collected on the day of TAVI, which served as the baseline. Key hemodynamic parameters including blood flow velocity, transvalvular pressure gradient, and left ventricular ejection fraction were measured at 30, 90, and 180 days postoperatively, in accordance with Valve Academic Research Consortium-3 (VARC-3), to evaluate temporal changes in hemodynamics. The effects of the AS model on valve function were further analyzed by integrating final histopathological findings and calcification degree outcomes.

**Results:**

Successful AS model establishment was achieved in all 11 sheep, with significant increases in mean transvalvular pressure gradient (Δ+17.98 ± 12.71 mmHg) and peak flow velocity observed post-modeling (Δ+ 2.23 ± 0.38 m/s). Post-TAVI evaluation demonstrated progressive hemodynamic normalization, achieving pre-modeling levels (1.72 vs. 6.91 mmHg, *P* = 0.058/0.80 vs. 1.51 m/s, *P* = 0.065) at 180-day follow-up. LVEF remained stable throughout the experimental period. Histopathological analysis indicated comparable calcification burden between 90 and 180 days (Calcium Content: *P* = 0.7459 and Calcification Score: *P* = 0.5455).

**Conclusions:**

The surgically induced ovine AS model effectively replicates clinically relevant hemodynamic perturbations while maintaining excellent procedural feasibility. TAVI in this model achieves complete hemodynamic normalization without accelerating bioprosthetic valve degeneration or calcification. This standardized preclinical platform enables rigorous evaluation of TAVI device performance and durability, providing robust scientific validation for translational applications.

## Introduction

Over the past decade, transcatheter aortic valve implantation (TAVI) has demonstrated significant clinical adoption due to its minimally invasive nature and accelerated postoperative recovery. This evolution has driven generational refinements in transcatheter valve systems (1–3). The technique employs endovascular delivery systems to facilitate percutaneous implantation of collapsible bioprosthetic valves via arterial access, circumventing age-related limitations of surgical aortic valve replacement. TAVI now represents first-line therapy for high-surgical-risk patients with complex comorbidities and inoperable cases of severe symptomatic aortic stenosis (4, 5).

Despite clinical success, preclinical validation faces persistent challenges. Reproducible large-animal models, mechanistic understanding of device-host integration, and standardized trial protocols constitute essential components of valvular therapy evaluation (6). Anatomically relevant preclinical models remain mandatory prior to human TAVI application. However, standard animal models lack calcific aortic valve pathology and stenotic annular substrates, impeding accurate replication of biomechanical environments critical to device deployment. This limitation compromises the reliability of biomechanical simulations and clinical translatability of findings (7).

Even if a pathological model of aortic stenosis can be successfully established, researchers face challenges in validating weather these models accelerate leaflet degeneration and calcification. This potential confounding factor may lead to misinterpretation of TAVI device efficacy during preclinical studies. The dual challenges of model development and validation persist despite advances in interventional cardiology. To address these limitations, this study aims to: (1) establish an ovine model of moderate-to-severe aortic stenosis using standardized aortic annuloplasty; (2) systematically assess the model's effects on the long-term functional stability of interventional valves, structural valve deterioration and calcification progression within a controlled experimental framework.

## Materials and methods

All experimental procedures were approved by the Institutional Animal Care and Use Committee (IACUC MFL-IACUC-2021-061) at GoldenWing Medical Technology Co., Ltd. (Suzhou, China), which adhered to Good Laboratory Practice standards. All animals underwent standardized perioperative management encompassing anesthesia protocols, postoperative care, scheduled follow-up monitoring, and humane endpoint euthanasia. Following terminal hemodynamic measurements, euthanasia was performed under deep general anesthesia, and a lethal intravenous bolus injection of potassium chloride (10%, 1.34 mEq/kg) was administered to ensure a painless and humane endpoint. Strict adherence to ethical requirements was maintained in accordance with Laboratory animal—Guideline for ethical review of animal welfare and ARRIVE 2.0 guidelines, ensuring ethical rigor and methodological transparency.

### Experimental design

Animals were assigned to two distinct cohorts: test and control groups. The test group (*n* = 11) underwent a standardized aortic annuloplasty (SAA) procedure under cardiopulmonary bypass (CPB). TAVI performed after 2–4 weeks, these modeled sheep were allocated into two subgroups for longitudinal tracking of valvular dynamics at 3-month (*n* = 6) and 6-month (*n* = 5) intervals. Additionally, a non-modeled control group underwent TAVI directly, with valve conditions thoroughly evaluated at the 3-month mark (*n* = 2) and 6-month interval (*n* = 1). All animals underwent transesophageal echocardiography (TEE) for final assessment prior to humane euthanasia.

### Animal preparation

Fourteen male sheep (12 months old; mean weight 58.7 ± 6.1 kg; provided by [Fulong Tengfei Laboratory Animal Research Institute, SCXK(Jing)-2018-0009] underwent standardized quarantine procedures including: (1) 7-day isolation with twice-daily veterinary inspections; (2) Following quarantine clearance, animals were transferred to AAALAC-accredited housing and acclimated for 21 days. Daily welfare assessments and weekly physical examinations by board-certified veterinarians confirmed preprocedural health stability.

### Bioengineered annular stent

**Figure 1:**
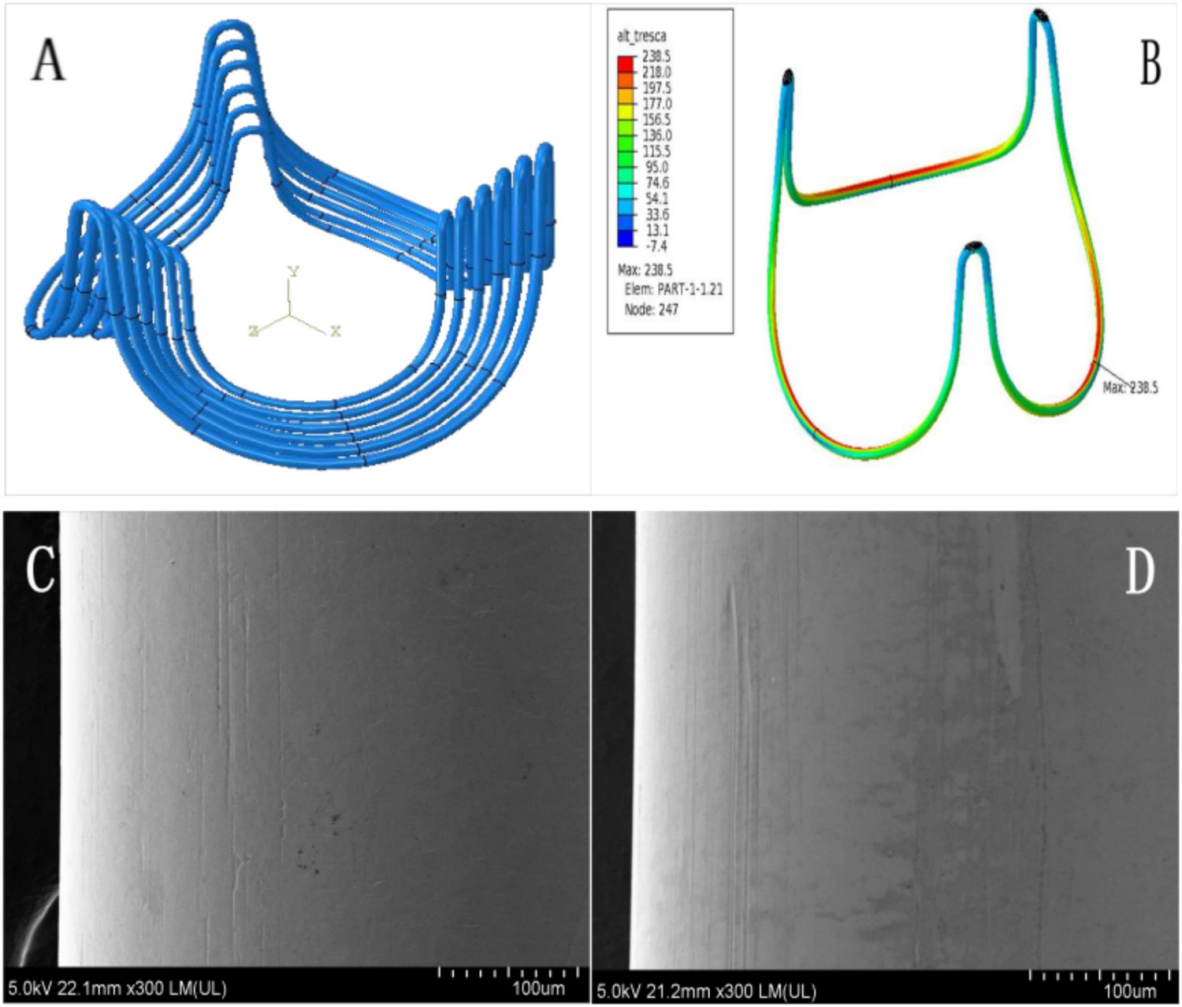
Characterization of bioengineered annular stent. To enhance bending accuracy and enable precise stress prediction, the model employs an 8-node hexahedral linear incompatible mode (curvature control: 0.02, size control: 0.10), with element dimensions meticulously configured at 0.06 mm **(A)**. The maximum stress concentration of annular sent manifests predominantly within the elliptical base's inner curvature zone **(B, highlighted in red)**. Comprehensive scanning electron microscopy analysis was performed at critical stress locations, comparing pre-fatigue conditions **(C)** with post-fatigue test results **(D)** under 300× magnification. This high-resolution comparison conclusively demonstrated no visible fatigue-induced surface damage on the annular stent.

The bioengineered annular stent, constructed from a CoCrNiMoFe alloy, was designed to align with aortic root geometry through an optimized streamline architecture. Its teardrop-shaped profile ensured physiological compatibility with aortic sinus, while the arched bridge-type structure with elliptical basal geometry promoted uniform stress distribution. The stent was encased in medical-grade knitted polyethylene terephthalate (PET) mesh to enhance biocompatibility. Structural validation, conducted via finite element analysis (FEA) per ISO 5840:2021 standards, confirmed fatigue resistance exceeding 4 × 10⁸ cycles, with critical stress distribution patterns presented (Figure 1).

### Establishment of as model

Anesthesia was induced intravenously with propofol (3–6 mg/kg) and intramuscular scopolamine (0.01 mg/kg). Following endotracheal intubation, sheep were positioned in right lateral recumbency. Inhalation anesthesia was maintained with isoflurane (1%–2%) in 100% oxygen. Surgical fields were shaved and prepared aseptically. Continuous monitoring included ECG, pulse oximetry, heart rate, invasive blood pressure, and arterial blood gas analysis. Prophylactic ceftiofur sodium (5 mg/kg IV) and lidocaine infusion (10 μg/kg/min IV) were administered. Full heparinization (heparin sodium 250 IU/kg IV) achieved target activated clotting time (ACT) ≥ 500 s.

A left thoracotomy was performed through the fourth intercostal space. The pericardium was incised and suspended for cardiac exposure. Normothermic cardiopulmonary bypass was established via descending aortic and right atrial appendage cannulation. Following cross-clamping of the ascending aorta, antegrade cold crystalloid cardioplegia was administered via direct infusion into the aortic root to induce cardiac arrest. Measurements included annular diameter, sinotubular junction dimensions, and sinus dimensions. A bioengineered annular stent was implanted beneath the native valve, positioned such that: (1) the stent base aligned with the annular plane connecting leaflet nadirs, and (2) three stent vertices corresponded to the valve commissures. Fixation utilized 5-0 polypropylene sutures at annular and commissural positions. Following de-airing, the aortotomy was closed with continuous suture and cross-clamp removed. Bypass was discontinued after stable rhythm recovery. The chest was closed with resorbable sutures following pleural drainage placement.

Postoperative recovery spanned 2–4 weeks under veterinary supervision, with appropriate analgesia and antibiotic prophylaxis. Pleural drains were removed based on clinical criteria.

### Transcatheter aortic valve implantation

Transcatheter aortic valve implantation procedure: The PerMedos valve system (currently in clinical trials) was deployed using a 22-French delivery system (capsule outer diameter: 7.3 mm; capsule length: 85 mm). Vascular access was achieved via a direct abdominal aortic approach rather than the transfemoral route. Specifically, a purse-string suture was pre-placed at the puncture site on the abdominal aorta. The aorta was then incised within this suture, the 22-Fr system was introduced, and the valve was deployed. Upon sheath removal, hemostasis was reliably achieved by tightening the pre-placed purse-string suture. Double-plane digital subtraction angiography (DSA) confirmed coplanar alignment of all three aortic sinuses. To prevent caudal migration, initial prosthesis positioning was established 3–4 mm above the annular plane. Real-time depth adjustment via the delivery system's push-pull mechanism maintained ventricular protrusion <6 mm. Post-deployment hemodynamic function was verified by pigtail catheter and orthogonal angiographic views (aortic root and left ventricular). In the experimental cohort, balloon predilation (1–2 inflations under 180–220 bpm rapid pacing; systolic pressure reduction to <60 mmHg) was selectively performed. Gradual stent expansion during rapid ventricular pacing (140–180 bpm) minimized device displacement during anchored geometry formation. The system permitted up to three positional recaptures for optimization before final release. Animals underwent stratified randomization. The experimental group received 24-mm valves allocated to 3-month (*n* = 6) or 6-month (*n* = 5) cohorts. Controls received 27-mm commercial bioprostheses (*n* = 3), with subgroups at 3-month (*n* = 2) and 6-month (*n* = 1) endpoints.

### Anticoagulation strategy

Upon successful validation of the aortic stenosis (AS) model, anticoagulation protocols were implemented according to standardized therapeutic monitoring. Modeled animals received dose-adjusted warfarin therapy (1–5 mg/day) targeting international normalized ratio (INR) values until 72 h pre-transcatheter aortic valve implantation, at which time they were transitioned to a combined warfarin-aspirin regimen. Post-TAVI antithrombotic management for both cohorts consisted of dual therapy initiation (warfarin 2.5 mg + aspirin 100 mg daily), with warfarin titration maintaining INR between 1.0–2.5 (1.5 × baseline) throughout the study duration. Dose adjustments followed predefined algorithms (INR <1.5: increase 10%–15%; INR >2.5: decrease 15%–20%) under weekly veterinary hematological monitoring until protocol-specified endpoints.

### Echocardiography

Following establishment of the AS model, comprehensive cardiac evaluations were performed using transesophageal echocardiography (TEE) at pre-TAVI baseline and postoperative day 30, 90, and 180. In compliance with VARC-3 guidelines (8), hemodynamic parameters including maximum velocity (Vmax), mean pressure gradient (MPG), left ventricular ejection fraction (LVEF), left ventricular end-diastolic dimension (LVEDD) and left ventricular end-systolic dimension (LVESD) were quantified. Additionally, valvular functional metrics were assessed, including leaflet mobility, valve regurgitation, paravalvular leakage and prosthesis morphological integrity through protocols pulsed-wave Doppler and color flow mapping.

### Histopathological examinations

The pathology team processed heart tissue samples using the established laboratory methods. Fresh specimens were stored at 4 °C, and formalin fixation was completed within 24 h of collection. For microscopic analysis, technicians used three staining methods: hematoxylin and eosin (H&E) for tissue structure, von Kossa staining for calcium detection, and Masson's trichrome for collagen visualization. Certified pathologists examined the prepared slides under microscopes using automated systems to ensure consistent results.

### Calcium quantitative analysis

At the study endpoint, three square tissue samples (5 mm × 5 mm) were collected from the central region of each prosthetic leaflet. The specimens were first digested in sterile polytetrafluoroethylene (PTFE) crucibles using a four-acid mixture containing nitric acid (HNO_3_), hydrofluoric acid (HF), perchloric acid (HClO_4_), and hydrochloric acid (HCl). Subsequently, 10 ml of aqua regia was added to achieve complete dissolution. After diluted to a final volume to 25 ml with deionized water, calcium levels were quantified through inductively coupled plasma atomic emission spectroscopy (ICP-AES). To ensure analytical reliability, all solutions underwent at least 3 h of sedimentation before spectral analysis to prevent instrument blockage.

### Statistical analysis

Statistical analyses adhered to differential distribution protocols: Normally distributed variables were expressed as mean ± SD, nonparametric data as frequency (percentage). Longitudinal echocardiographic parameters in the modeling cohort were analyzed using one-way repeated measures ANOVA with Greenhouse-Geisser correction for temporal comparisons, while intergroup differences employed independent Student's *t*-tests with Welch's correction. Distribution normality was verified through Kolmogorov–Smirnov testing, with non-conforming datasets subjected to Mann–Whitney *U*-tests. The statistical significance threshold was set at *α* = 0.05 (two-tailed). All computational workflows, including data visualization, were executed in GraphPad Prism 10.

## Results

All experimental sheep completed the protocol per study design. In the model cohort (*n* = 11), surgically created aortic stenosis was successfully established prior to TAVI, while three control animals received direct TAVI without antecedent pathology induction. Postoperative survival analysis documented five model animals reaching scheduled endpoints at 90 days and six at 180 days, compared to two and one control animals at respective timepoints. No perioperative mortality or severe procedure-related vascular complications occurred during observation.

### Echocardiography

All surgical interventions successfully induced aortic stenosis (AS) models in 11 sheep. Postprocedural echocardiography immediately conducted demonstrated statistically significant elevations in peak aortic forward flow velocity (0.82 ± 0.12 m/s vs. 3.05 ± 0.34 m/s, *P* < 0.001) and mean transvalvular pressure gradient (1.71 ± 1.00 mmHg vs. 19.69 ± 2.88 mmHg; *P* < 0.001), confirming acute hemodynamic alterations. These parameters surpassed the diagnostic thresholds for moderate-to-severe aortic stenosis (peak velocity ≥3 m/s) under current valvular pathology standards. This alignment conclusively validated the successful attainment of the predefined modeling objectives.

**Figure 2:**
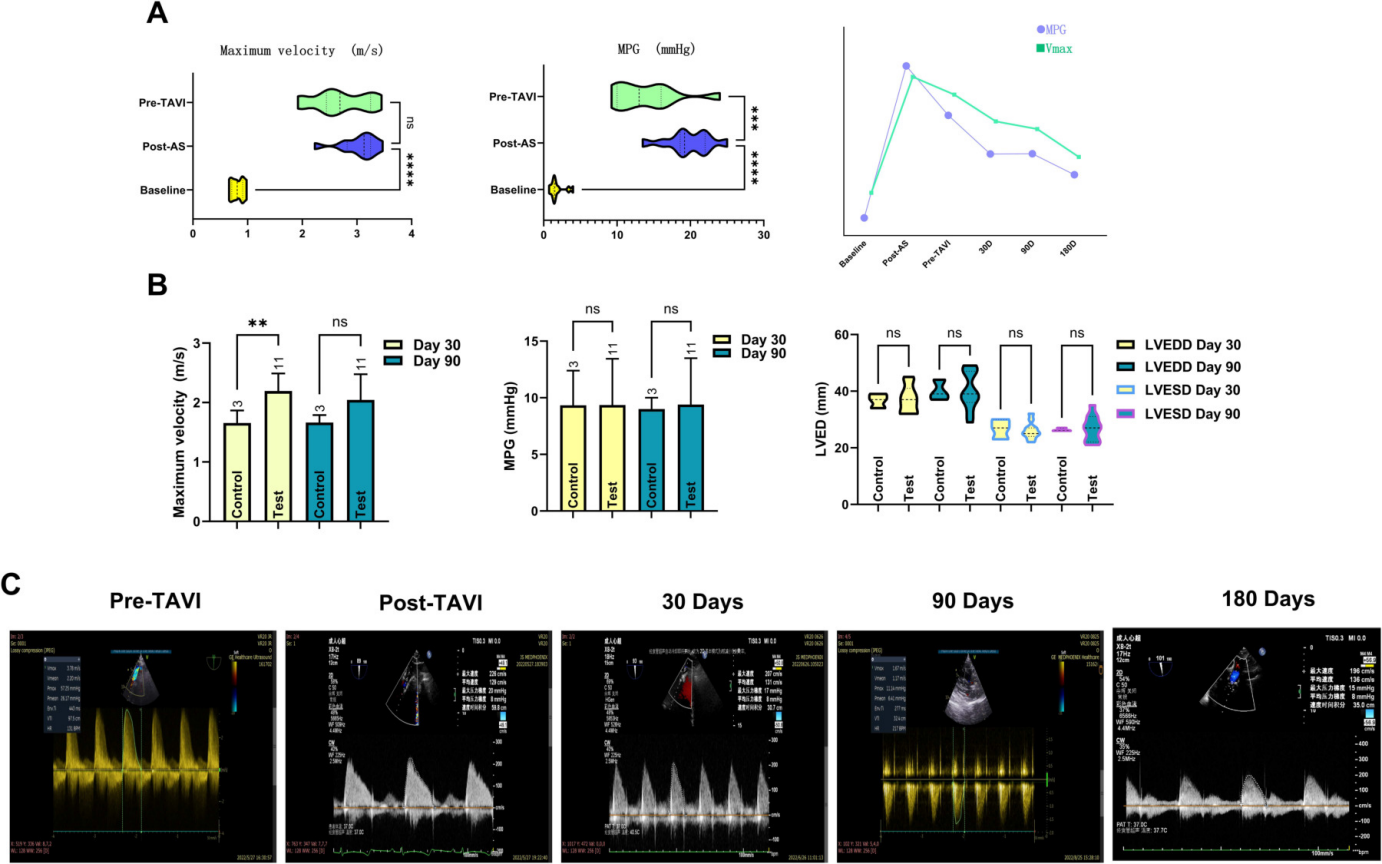
Documentation of echocardiographic changes across the full trial duration. Following establishment of the AS model, both Vmax and MPG demonstrated significant changes. However, only Vmax exhibited sustained stability prior to TAVI onset. Temporal slope analysis revealed MPG declined at a significantly faster rate than Vmax. This divergence originates from Vmax's heightened sensitivity to the anatomical severity of aortic stenosis itself, whereas MPG elevation primarily reflects increased resistance caused by restricted leaflet mobility, an effect not solely attributable to the stenotic orifice. The line chart further illustrates nuanced mechanistic distinctions governing their pre-TAVI evolutionary trajectories **(A)**. The transient disparity in Vmax observed in the control group at 30 days was attributable to larger device sizes; as the experimental group's Vmax progressively decreased, both groups achieved hemodynamic equivalence by day 90. Comparative analysis showed no statistically significant differences in cardiac anatomical metrics or MPG between groups **(B)**. Spectral Doppler data clearly demonstrate the temporal changes in peak velocity (Vmax) and MPG within the stenosis model, documenting its progression from moderate-severe aortic stenosis to a physiologically normal state **(C)**.

Cardiac functional parameters demonstrated that the model induced left ventricular dysfunction within 2–4 weeks post-surgery, as evidenced by a significant reduction in LVEF (from 68.61 ± 9.77 at baseline to 54.87 ± 6.32, *P* < 0.001). In contrast, left ventricular dimensions remained unaffected by the procedure, with no significant changes observed in LVSDD or LVEDD. In the 3-month cohort, post-TAVI TEE showed stable and not significantly changed peak flow velocities and transvalvular gradients at 30- and 90-day assessments. In contrast, the 6-month cohort demonstrated gradual hemodynamic normalization, achieving optimized parameters by the 180-day evaluation, as evidenced by a significant reduction in peak flow velocity (*P* < 0.001) and mean transvalvular pressure gradient (*P* < 0.005) compared to pre-TAVI levels. Meanwhile, the LVEF was nearly restored to the baseline level (*P* = 0.949). All prostheses demonstrated robust functional integrity throughout the study period, exhibiting no severe stenosis, clinically significant regurgitation, or major paravalvular leakage (PVL). Quantitative analysis identified three mild PVL cases (VARC-3) in the 6-month cohort. Two persisted at 180 days, while one resolved completely by the endpoint. This contrasted with the 3-month cohort, where two mild PVL cases were observed at the 90-day evaluation. Regurgitation profiling revealed three instances of mild aortic valve regurgitation in the 6-month group, while the 3-month group showed one moderate mitral regurgitation. Intriguingly, control animals displayed a solitary persistent mild mitral regurgitation within the 6-month subgroup, with all other echocardiographic metrics aligning within expected physiological ranges (Figure 2 and Table 1).

**Table 1:** Temporal TEE and Doppler evaluation.

Indicators	Baseline (*n* = 11)	Post-AS (*n* = 11)	Pre-TAVI (*n* = 11)	*P*-value (Pre-TAVI vs. baseline)	30-day follow-up (*n* = 11)	90-day follow-up (*n* = 11)	180-day follow-up (*n* = 5)	*P*-value (180-day follow-up vs. pre-TAVI)
Doppler measurements								
Vmax (m/s)	0.82 ± 0.12	3.05 ± 0.34	2.97 ± 0.52	0.0001	2.19 ± 0.29	2.04 ± 0.43	1.51 ± 0.25	0.0001
MPG (mmHg)	1.80 ± 1.00	19.78 ± 3.13	13.93 ± 4.32	0.0001	9.15 ± 3.93	9.75 ± 3.57	6.91 ± 2.86	0.0059
LVEF (%)	68.61 ± 9.77	64.72 ± 5.31	54.87 ± 6.32	0.004	61.28 ± 9.43	57.85 ± 9.35	64.66 ± 5.75	0.259
LVEDD (mm)	38.64 ± 4.97	39.73 ± 3.07	39.55 ± 6.52	0.994	37.27 ± 4.65	40.18 ± 6.73	37.64 ± 4.27	0.852
LVESD (mm)	23.45 ± 3.14	25 ± 2.72	26.09 ± 5.97	0.440	26.00 ± 2.93	26.82 ± 4.41	23.6 ± 3.05	0.544
Echocardiographic findings (*n*)								
Vmax < 3.0 m/s	11	3	5		11	11	11	
MPG > 20 mmHg	0	0	0		0	0	0	
Small calcifications on leaflets	0	0	0		0	0	0	
Perivalvular leak	0	0	0		2	2	2	
Mild aortic regurgitation	0	11	11		2	2	2	
Moderate aortic regurgitation	0	0	0		0	0	0	
Mild mitral regurgitation	0	0	0		1	1	1	
Moderate mitral regurgitation	0	0	0		0	0	0	

### Digital subtraction angiography

**Figure 3:**
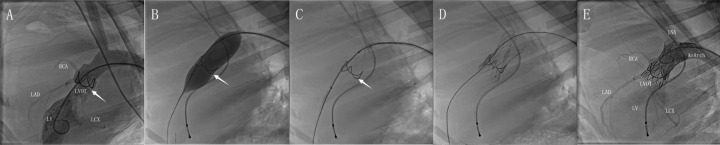
DSA for the full-lifecycle evolution of model groups. Post-implantation assessment demonstrated favorable morphological adaptation of the annular stent. Its elliptical basal segment resided within the valve sinus **(A, arrow)**. Angiography confirmed unimpaired coronary perfusion without pathological regurgitation (Please also refer to V1 in the supplementary materials). Pre-TAVI targeted balloon pre-dilation elicited controlled basal expansion with observable morphological changes **(B)**. This configuration persisted post-expansion, featuring a widened angle in the bridge-shaped arch structure that optimized subsequent TAVI delivery **(C)**. Following deployment, the prosthesis achieved stable integration with full expansion of the left ventricular outflow tract stent and clear visualization of radiographic markers **(D)**. Post-procedural aortography demonstrated preserved bilateral coronary flow and absence of periprosthetic contrast leakage around valve leaflets or anchoring components. The distal stent remained firmly compressed between the innominate artery and aortic arch curvature, without affecting vascular morphology **(E)**.

Digital subtraction angiography (DSA) analysis revealed stable annular fixation of the bioengineered annular stent in modeled animals, with three-dimensionally curved force transmission surfaces effectively preventing coronary ostium obstruction (Supplementary Video V3). The overall structure of the stent revealed seamless anatomical compliance with aortic root kinematics, faithfully preserving the native leaflet coaptation mechanics through precision-engineered motion alignment. Post-TAVI imaging demonstrated secure device anchoring at the left ventricular outflow tract-aortic annulus interface, with preserved patency of bilateral coronary ostia (Supplementary Video V2). The distal crown exhibited optimal physiological positioning between the origin of the brachiocephalic artery and the curvature of the aortic arch (Figure 3). Control group prostheses likewise achieved meticulous annular seating, maintaining preserved leaflet kinematics throughout cardiac cycles while demonstrating complete absence of paravalvular leakage, thereby confirming consistent deployment accuracy across all cases.

### Anatomic findings

**Figure 4:**
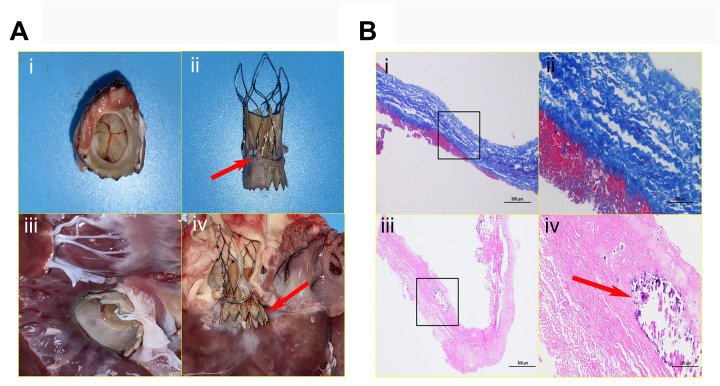
Anatomical characteristics of devices and histopathological examination. The interventional valve exhibits an inherent parabolic morphology on its inflow surface, characterized by three fully intact, seamlessly coapting leaflets **(A-i)**. The presence of excellent stent positioning within the annular framework (arrow), exhibiting complete endothelial coverage and preserved structural integrity **(A-ii)**. The outflow surface clearly reveals preserved physiological angulation, meticulously preserving the integrity of the mitral valve apparatus, including the anterior leaflet and chordae tendineae free from any mechanical deformation; complete endothelialization is clearly evident over the LVOT segment **(A-iii)**. This image vividly demonstrates pronounced axial valve displacement protruding into the LVOT (arrow), causing a functional compromise of the mitral valve's anterior leaflet closure mechanism and culminating in moderate regurgitation (A-iv). **(B)**. Collagen fibers exhibit an orderly arrangement, homogeneous staining, and no obvious fractures (blue-stained areas). A thin layer of eosinophilic, red-stained fibrin adheres to both leaflet margins; no significant endothelial cell coverage is present, and inflammatory cell infiltration within the leaflet is absent. Alizarin red staining reveals a relatively thick fibrin layer on both sides of the prosthetic valve, with calcification foci (arrow) visible at the leaflet edges and within the adherent fibrin.

All experimental animals were humanely euthanized through terminal anesthesia, supplemented by intravenous administration of heparin sodium and potassium chloride. Subsequent six-month follow-up in the model cohort revealed seamless structural integration between the implanted prostheses and stent, evidenced by complete endothelial encapsulation and preserved symmetrical coaptation of all three leaflets. Valve kinematics exhibited precise physiological opening angles, featuring smoothly contoured parabolic inflow surfaces while showing no signs of pathological remodeling. Mitral apparatus integrity remained intact, preserving the intricate spatial relationships between anterior leaflets and chordal structures. Longitudinal surveillance revealed a singular instance of prosthetic axial migration at the 3-month follow-up, resulting in functional interference with mitral leaflet mobility. All other indicators remained closely aligned with the six-month observation findings, as illustrated (Figure 4A).

### Pathological characteristics in valve leaflets

Masson's trichrome staining revealed negligible fibrous tissue formation at the base of the porcine pericardial prosthetic valve. Uniformly deposited fibrin was noted on both leaflet surfaces. Collagen fibers demonstrated clearly defined orientation, structural preservation, and continuous arrangement. High-power microscopic examination detected no significant inflammatory cell infiltration. The valve base-tissue interface exhibited confluent endothelial cell coverage. Focal areas of early hyaline degeneration were present at the free leaflet margins, without structural thickening or contracture (Figure 4B).

### Calcification outcomes in valve leaflets

We conducted a comprehensive analysis of quantitative calcium deposition patterns and calcification scores across 11 experimental subjects. The findings revealed remarkably consistent calcification profiles between the 90-day and 180-day cohorts within this model framework, with intergroup comparisons showing no statistically significant divergence. Longitudinal observation through 180 days demonstrated no detectable acceleration in calcification progression. Valvular leaflet calcium quantification revealed non-significant mineralization levels (<1% of leaflet dry mass) in all specimens, corroborated by histopathological verification of preserved collagen fiber architecture with sporadic microcalcific foci, as detailed in Table 2.

**Table 2:** Quantitative detection of calcium content in valve leaflets.

Group	Calcium content (μg/mg)	Calcification score
90-day follow-up	0.09	0
	0.084	0
	0.176	0
	0.045	0
	0.165	0
	9.80	1
180-day follow-up	6.60	1
	5.40	1
	0.177	0
	0.080	0
	0.079	0
*P*-value	0.746	0.546

### Changes in renal function

**Figure 5:**
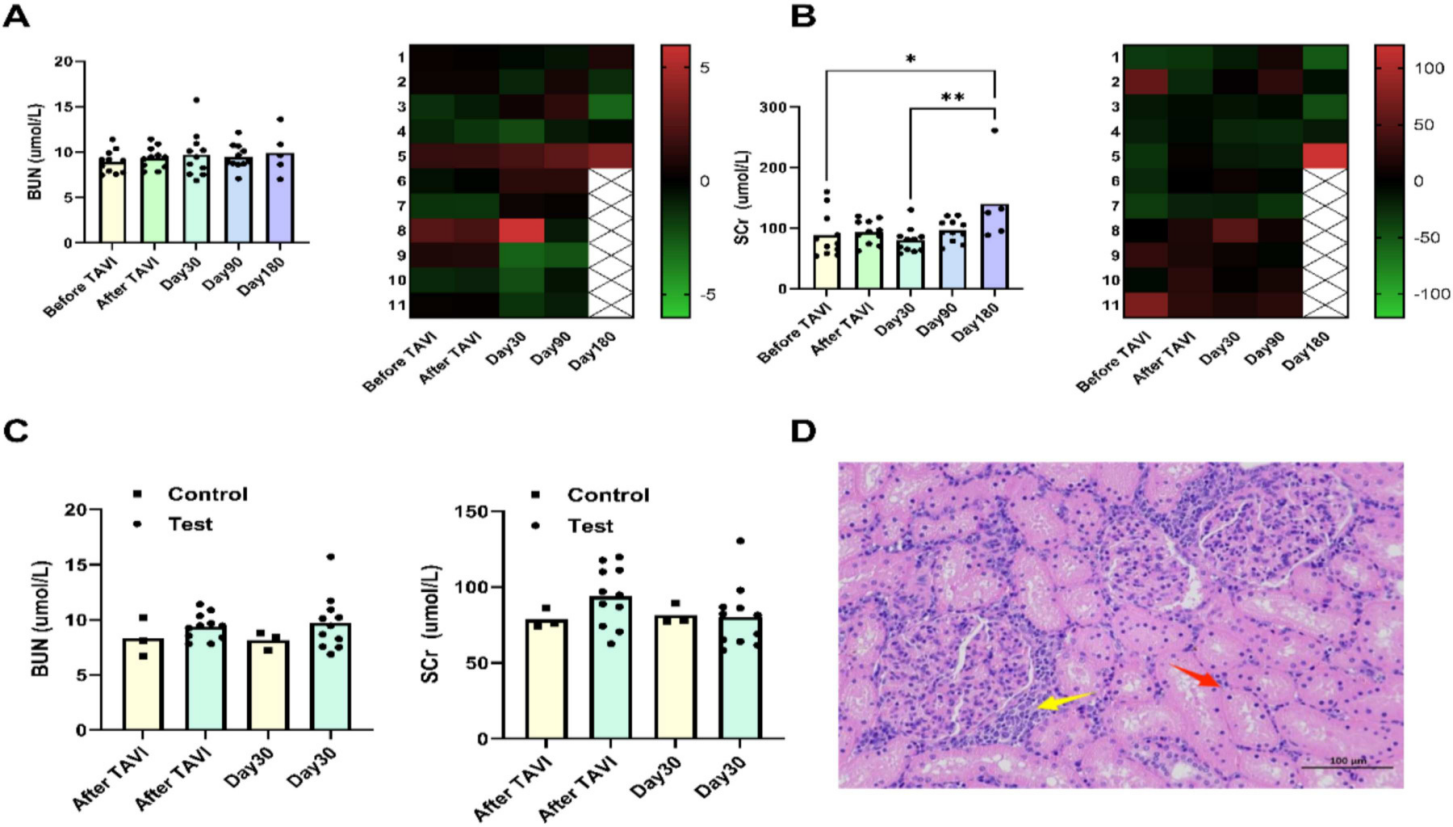
Assessment of kidney function. BUN levels remained stable throughout follow-up, showing no statistically significant fluctuations at any interval. In the 180-day subgroup, individual sheep exhibited elevated SCr concentrations, exceeding preoperative baselines **(A,B)**. Pathological analysis of kidney tissue from this animal revealed: renal tubular epithelial cells displayed mild vacuolar degeneration **(D)**, with focal degeneration and necrosis (red arrows), and multifocal lymphocyte infiltrates (yellow arrows) in interstitial regions. Compared to controls, neither BUN nor SCr showed clinically significant changes after TAVI or during 30-day postoperative assessments **(C)**.

To mitigate potential contrast agent overdose risks arising from technical complexities in TAVI procedures for aortic stenosis models, this study instituted rigorous renal function monitoring protocols. By systematically documenting contrast agent volumes administered to experimental animals pre- and post-operation, while concurrently tracking fluctuations in two critical renal biomarkers, serum creatinine (SCr) and blood urea nitrogen (BUN), we executed a comprehensive longitudinal evaluation. Experimental analyses demonstrated no statistically meaningful variation in contrast agent utilization between the model development cohort and the TAVI direct intervention cohort. Crucially, comparative assessments of postoperative renal biomarkers, including SCr and BUN trajectories, revealed that intergroup disparaties failed to attain statistical significance (Figure 5).

### Anticoagulation use

Therapeutic anticoagulation, targeting a range of 1.0–2.5 (INR), was rigorously sustained throughout the entire postoperative survival phase in all experimental animals. Both model and control cohorts demonstrated equivalent anticoagulation profiles at terminal endpoints, with absence of anticoagulant-related hemorrhagic complications, confirming adherence to the validated therapeutic window.

## Discussion

Preclinical validation of TAVI devices in large-animal models constitutes a critical step preceding clinical trials. Comprehensive evaluation of device durability, calcification resistance, and antithrombogenic properties before human application substantially mitigates clinical risks (9–11). However, existing models fail to faithfully replicate the pathoanatomic features of calcific aortic stenosis, typically requiring device implantation in non-diseased aortic roots. This approach induces several limitations: the elastic, non-calcified annulus provides insufficient resistance to the stent's radial expansion force, potentially leading to device migration, malposition, or paravalvular regurgitation, thereby compromising assessment of anchoring mechanisms and long-term performance (12, 13). Furthermore, creation of moderate-to-severe aortic stenosis models necessitates substantial procedural expertise with extensive optimization, while acute afterload elevation frequently causes perioperative mortality - imposing significant resource consumption and ethical concerns (14).

Recent collaborative research has yielded innovative large-animal models of mechanically induced aortic stenosis. These efforts implemented diverse methodologies including: cardiopulmonary bypass-assisted surgical implantation, heterotopic pulmonary artery/thoracic aorta delivery, valve-in-valve deployment, aortic banding, and transcatheter insertion in non-stenotic annuli. Although providing anchoring segments for TAVI, these models failed to generate clinically significant moderate-to-severe hemodynamic alterations (15, 16). Additionally, non-standardized implantation devices frequently induced mild-to-moderate aortic regurgitation, compromising model validity. Crucially, these models lack controlled validation regarding stenosis-induced effects on long-term bioprosthetic valve properties. A single study establishing severe stenosis via surgical intervention exclusively evaluated coronary physiology and microvascular function, omitting assessment of TAVI outcomes (17).

In exploring aortic stenosis modeling strategies, in silico simulation technologies provide significant advantages by replicating stenosis-associated hemodynamics while circumventing ethical and technical constraints of animal experiments (18). These methods employ patient-derived ultrasound/CT data to construct parameterized geometric models, utilizing finite element analysis (FEA) for tissue mechanics simulation and reverse calcification technology (RCT) for calcification progression reconstruction. Fluid-structure interaction (FSI) analysis integrates computational fluid dynamics (CFD) to characterize hemodynamic alterations across stenosis severities (19, 20). Soft robotic sleeve technology incorporates pneumatic actuators encircling the ascending aorta to dynamically modulate pressure, accurately replicating hemodynamic profiles including vortex dynamics and energy dissipation under real-time MRI monitoring (21). Artificial intelligence-driven multimodal modeling (DeepCarve) combines CFD with 3D printing to generate biohybrid systems, automating computational mesh generation and enabling real-time in silico hemodynamic validation (22). However, despite high-fidelity replication of luminal 3D morphology and acute hemodynamic parameters, in silico models exhibit limited capacity for simulating chronic pathological processes such as endothelial remodeling and progressive calcification (23). Current systems also fail to reproduce complex device-tissue mechanical interactions or temporal biointerface adaptation—key mechanisms underlying TAVI complications including paravalvular leakage and stent migration (24). These constraints underscore the essential role of animal models in long-term biological evaluation.

Given the limitations of existing *in vivo* and in silico models, we established a standardized ovine model of aortic stenosis through annular constriction combined with bioengineered stent technology. This model accurately recapitulates the pathomorphological environment of human aortic valve stenosis, providing a preclinical platform for evaluating TAVI devices under hemodynamically relevant conditions. Furthermore, the bioengineered annular stent anatomically simulates surgical bioprosthetic valve frames, enabling evaluation of valve-in-valve implantation scenarios. Unlike prior approaches, this model achieves clinically significant moderate-to-severe stenosis hemodynamics, permitting TAVI deployment under high-velocity flow with real-time functional monitoring (25). Based on the experimental findings of this study, we conclude that modifying the aortic annulus diameter can induce clinically significant aortic stenosis in the short term, with its effects swiftly manifesting as measurable alterations in blood flow velocity. In contrast, approaches aimed at elevating the transvalvular pressure gradient typically necessitate leaflet calcification—a process that results in gradual hemodynamic shifts rather than the immediate changes observed with aortic annular constriction (26). Thus, our method exhibits superior clinical utility for establishing precise aortic stenosis models. In conclusion, leveraging our aortic stenosis model, we meticulously tracked bioprosthetic valve performance and histobiological processes through 3–6month longitudinal observations paired with cross-sectional analyses. Our analysis demonstrates that this model maintains hemodynamic stability for 2–4 weeks post-surgery, effectively simulates aortic stenosis for TAVI implantation, and does not adversely affect the long-term condition of the leaflet tissues. Notably, our results indicate that left ventricular function declines due to the physical stenosis. We hypothesize that a prolonged waiting period would risk inducing severe ventricular hypertrophy or even irreversible heart failure. Such a complex and unstable disease state would be difficult to control and would introduce confounding variables. Therefore, by intervening within the 2–4-week window, we maintained a clear and controllable pathological model while ensuring procedural feasibility. Future studies may explore the potential of this model beyond this timeframe. The results of these efforts will establish critical data support for future preclinical TAVI validation work.

This pilot study demonstrates the translational utility of a SAA model for evaluating TAVI hemodynamics. The bioengineered annular stent demonstrates long-term stability, effectively preserving structural integrity without accelerating valve degeneration or calcification. However, a critical distinction persists between geometrically uniform, symmetric structures and the clinically observed irregular spatial configurations of calcified nodules. This structural variance may result in an overestimation of anchor devices' fixation efficacy within standardized models, attributable to their inherently stable stress distribution patterns. To bridge this gap, future iterations must integrate calcific nodule heterogeneity. Leveraging AI and subtractive 3D bioprinting of CT-derived calcification maps could enable Grade 4 aortic calcification replication—a pivotal advancement for robust TAVI assessment and accelerated therapy development.

## Limitations

While this study pioneers a standardized ovine aortic stenosis model with validated hemodynamic reproducibility, four methodological constraints warrant consideration: (1) The control group required oversized device for implantation, making it impossible to ensure that subsequent complications and long-term survival rates could be adequately managed. Following a thorough evaluation of cost-effectiveness and adherence to ethical guidelines, a minimal sample size was selected for the trial cohort (*n* = 3) - a methodological concession that may inadvertently compromise the statistical validity of comparative analyses across experimental groups. (2) The protocol requires extended cyclic fatigue validation. System-level durability testing, particularly multi-month accelerated wear simulations, would further prolong preclinical validation. (3) Validation solely centered on self-expanding transcatheter systems overlooks balloon-expandable counterparts, which may constrain generalizability to heterogeneous TAVI device platforms. (4) While the simulation protocol exhibited high-fidelity replication of aortic calcific annulus topography, the protocol failed to recapitulate the biomechanical mechanisms underlying pathomechanical leaflet calcification. (5) The present study did not include a stent-only control group, as the primary objective was to establish a standardized and hemodynamically stable pathomimetic platform for TAVI evaluation, which required progression to valve implantation in all test animals. Therefore, the natural history of the induced stenosis and its specific impacts on ventricular remodeling in the absence of TAVI intervention remain to be elucidated in future studies. (6) Hemodynamic assessment relied solely on non-invasive transesophageal echocardiography. Although this approach ensured consistent longitudinal data acquisition, the absence of simultaneous invasive pressure measurements, precludes direct validation against the gold-standard for gradient assessment. Future studies will incorporate such synchronous measurements to provide a more rigorous hemodynamic characterization.

## Conclusion

SAA ovine models provide an anatomically congruent platform for TAVI device evaluation, enabling systematic safety and efficacy profiling while mitigating translational risks through rigorous validation. However, achieving pathologically validated annular calcification and hemodynamically significant stenosis demands further precision optimization. Such advancements would establish physiologically relevant benchmarks for next-generation TAVI device development.

## Data Availability

The original contributions presented in the study are included in the article/Supplementary Material, further inquiries can be directed to the corresponding authors.
